# Journalistic Notes

**Published:** 1891-03

**Authors:** 


### Journalistic Notes.

The *Neurologisches Centralblatt*, published under the direction of Prof. Mendel, of Berlin, begins the new year under most favorable auspices. It is devoted entirely to neurology and psychiatry, and keeps its readers well informed on the advances continually taking place in these important fields. Its last number contains an abstract from the proceedings of the Buffalo Pathological Society.

---

The New Year brings *The Microscope*, edited by Dr. Alfred C. Stokes, of Trenton, N. J., greatly improved, to our exchange table. New type and appropriate head pieces and vignettes add much to make it a credit to the typographical art. *The Microscope* is always overflowing with the best of literature in its special line, and its appearance is always looked forward to with genuine pleasure.

A NEW journal, *Anales de la Asistencia Publica*, published at Buenos Ayres, and as its title indicates devoted to Hygiene, has recently made its appearance upon our exchange table. It is a monthly publication, large size, of excellent paper and print, and devoted more to the questions of public health and hygiene. Among its first contributions are the Sanitary Condition of Rio Janeiro and articles on the Treatment of Syphilis; Report on the Influenza in Argentina, Year 1890; Meteorology and Hygiene, etc. The subscription price is one Peso, and is published at Corrientes, 829 Buenas Airas.

---

THE *Revista De Ciencias Medicas De Barcelona* begins its seventeenth year with a new garb and new form, thus materially increasing its attractiveness. The *Revista* is one of the best of our Spanish exchanges, and is in the front rank of medical journalism. It stands aloof from that class of journals which compel their readers to dissect out the reading matter from a mass of misplaced advertisements.

